# The phyllosphere microbiome of host trees contributes more than leaf phytochemicals to variation in the *Agrilus planipennis* Fairmaire gut microbiome structure

**DOI:** 10.1038/s41598-021-95146-9

**Published:** 2021-08-05

**Authors:** Judith Mogouong, Philippe Constant, Pierre Legendre, Claude Guertin

**Affiliations:** 1 Institut National de la Recherche Scientifique, Centre Armand-Frappier Santé Biotechnologie, Laval, QC H7V 1B7 Canada; 2 Département de Sciences Biologiques, Université de Montréal, C.P. 6128, succ. Centre-ville, Montréal, QC H3C 3J7 Canada

**Keywords:** Invasive species, Microbial ecology, Microbiome

## Abstract

The microbiome composition of living organisms is closely linked to essential functions determining the fitness of the host for thriving and adapting to a particular ecosystem. Although multiple factors, including the developmental stage, the diet, and host-microbe coevolution have been reported to drive compositional changes in the microbiome structures, very few attempts have been made to disentangle their various contributions in a global approach. Here, we focus on the emerald ash borer (EAB), an herbivorous pest and a real threat to North American ash tree species, to explore the responses of the adult EAB gut microbiome to ash leaf properties, and to identify potential predictors of EAB microbial variations. The relative contributions of specific host plant properties, namely bacterial and fungal communities on leaves, phytochemical composition, and the geographical coordinates of the sampling sites, to the EAB gut microbial community was examined by canonical analyses. The composition of the phyllosphere microbiome appeared to be a strong predictor of the microbial community structure in EAB guts, explaining 53 and 48% of the variation in fungi and bacteria, respectively. This study suggests a potential covariation of the microorganisms associated with food sources and the insect gut microbiome.

## Introduction

The study of environmental microbial communities may contribute to a deeper understanding of an ecosystem, particularly where their interactions with abiotic and biotic factors are considered. Living organisms are colonized by various microorganisms, encompassing bacteria, fungi, archaea, and protozoa^1^ that significantly participate in the host’s essential physiological functions. The combination of compositional information about the microorganisms inhabiting a host and their genomes is defined as the host microbiome. Insects are commonly used as model systems for microbiome studies, and the microorganisms colonizing them are considered primarily symbiotic as they are closely involved in the performance of their host’s essential ecological functions as well as their biological functions and fitness^2^. They are considered model systems because their gut has a lower diversity microbiome than mammalian systems, allowing cost-effective and time-efficient studies to investigate the complexity of microbial interactions^3^. There is a growing body of evidence that insect gut microbiomes are shaped by variables such as the developmental stage^4,5^, diet^6–8^, environment^9^, plant defense mechanisms^10^, and even the insect population density^11^. Several research efforts investigated the relationship between pest management and the insect microbiome, some suggesting microbiome manipulations^3^. One approach example based on replacing the primary symbiont *Buchnera* with a specific genotype by microinjection in the pea aphid could alter the thermal tolerance of the insect^12^. The potential contributions of the insect microbiome to diverse survival processes, including those related to its invasiveness traits, need to be addressed for efficient pest management approaches. Although studies of microbiome dynamics and plasticity have been increasing in number, most of them suffer from limitations including lack of a holistic approach for elucidating the mechanisms of complex ecological processes. One of the most frequently used analyses to investigate hypotheses related to the microbiome is redundancy analysis (RDA), a form of canonical analysis that is part of the regression modelling family. RDA is well suited for investigating variations in a response data matrix by one or several explanatory matrices. In addition, partial RDA allows researchers to identify redundancies among sets of explanatory variables. In this study, redundancy analysis is the computational workhorse in the variation partitioning method proposed by Borcard, et al.^13^ and Peres-Neto, et al.^14^ to determine the independent and joint contributions of multiple explanatory datasets and their redundancy in explaining host-microbiome variations. This approach is expected to improve understanding of the relationship between the host microbiome and the environment and help identify environmental predictors that explain the structure and composition of those microbial communities.

Among the Insecta, some species are considered threats to an ecosystem when they cause severe plant damage, especially to forest trees like the American ash. The emerald ash borer (EAB), *Agrilus planipennis* Fairmaire, is a holometabolous insect reported to cause significant environmental and economic damage to several *Fraxinus* species in North America^15–17^. After its introduction, EAB developed into an invasive pest in the highly urbanized area of Detroit, Michigan, USA^18^. As the life traits of EAB make the early detection very difficult, it continues to spread across the continent and is responsible for millions of ash tree’s death^19^. That insect colonizes health and stressed ash trees and has been reported to be a disruption cause for natural processes threatening some native ash trees and, consequently, the forest diversity^20^. Moreover, several studies have shown that ash mortality can impact the hydrology in wetland and harm insect species or fauna reported to be ash dependant^21,22^. Despite all the control strategies implemented, including biological control and the introduction of natural enemies that contribute to slow the spread of EAB, it is almost impossible to stop its progression. During the host establishment, herbivorous insects may have to cope with defensive plant responses, mainly the production of molecules harmful to insects and the intensification of the lignification process^23–25^, and with microorganisms inhabiting the leaves of the host tree. Insects harbour many different biotopes in their bodies, but the gut is the most favourable one for colonization by microorganisms^26^. The gut is largely a protected environment for microorganisms, but it can present them with some adverse conditions, such as harmful phytochemicals and other factors shaping microbial community structure. Similarly, the phyllosphere, represented by the aerial part of the leaves (including endophytes and epiphytes)^27^, can be associated with some microorganisms, which may help the plant facing pathogens. Although many studies reported on the effects of insect gut-associated microorganisms on leaf defences^28^, the relationship between environmental microorganisms and the insect gut microbiota has received little attention. As the microbiome may contribute to the insect invasiveness processes, a better comprehension of its plasticity mechanisms based on a holistic approach should open new research avenues valuable for pest management. Since recent decades, that research field is getting more attention with some strategies targeting microbiome manipulation approaches that could alter the pest traits^3^. Our previous works^11^ showed that some changes in the taxonomical structure of the bacterial community associated with the adult EAB gut could be related to the level of infestation of the host tree, suggesting that there may be a bipartite relationship between the “adult EAB gut microbiome” and its “host tree properties including their physiological traits”. This study was designed to test the hypothesis that variation in the insect gut microbiome can be attributed to host tree leaf phytochemicals, the phyllosphere microbiome, and geographical location. A field survey was conducted to address three complementary questions: (1) How are the microbial communities structured in the adult EAB gut and in the phyllosphere biotope (similarities and differences)? (2) Could the ash leaf microbiome and the phytochemical profile be predictors of the bacterial and fungal communities associated with adult EAB gut? And (3), to what extent do ash leaf microbiome and phytochemicals explain variations in the adult EAB gut microbiome? We compared the microbial communities of the insects and the host trees, and performed a series of redundancy analyses to identify potential predictors among host tree leaf phytochemicals, phyllosphere microbiome, and geographic location for variations in the EAB gut microbiome. Selected predictors were then used to partition the variations observed within the adult EAB gut microbial communities. The individual and redundant contributions of explanatory matrices were computed to model the observed variations in EAB gut microbiome structure.

## Results

**Figure 1:**
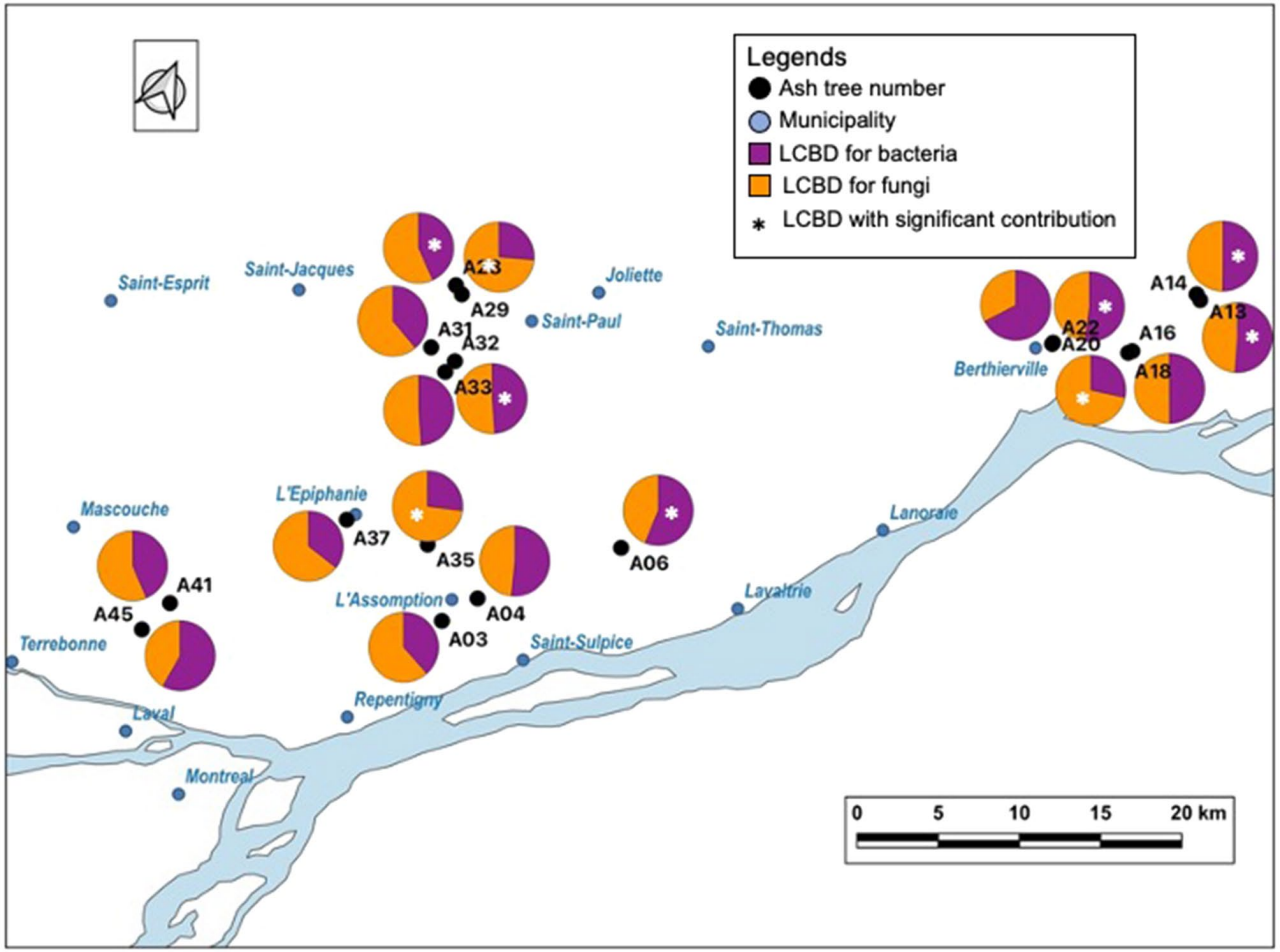
Map representing sampling sites including the LCBD value per site. For each site LCBD values of bacteria (purple) and fungi (orange) are represented in percentages illustrated in a pie chart. The white star on the colour indicates the significant contribution of the corresponding community to the local β-diversity. Thus, the bacterial community was found significantly contributing to the local β-diversity in six sites (A06, A13, A14, A22, A28, and A32), Holm-corrected LCBD *p* values, whereas the fungal community was found significantly contributing to the local β-diversity in three sites (A18, A29, and A35), Holm-corrected LCBD *p* values.

**Figure 2:**
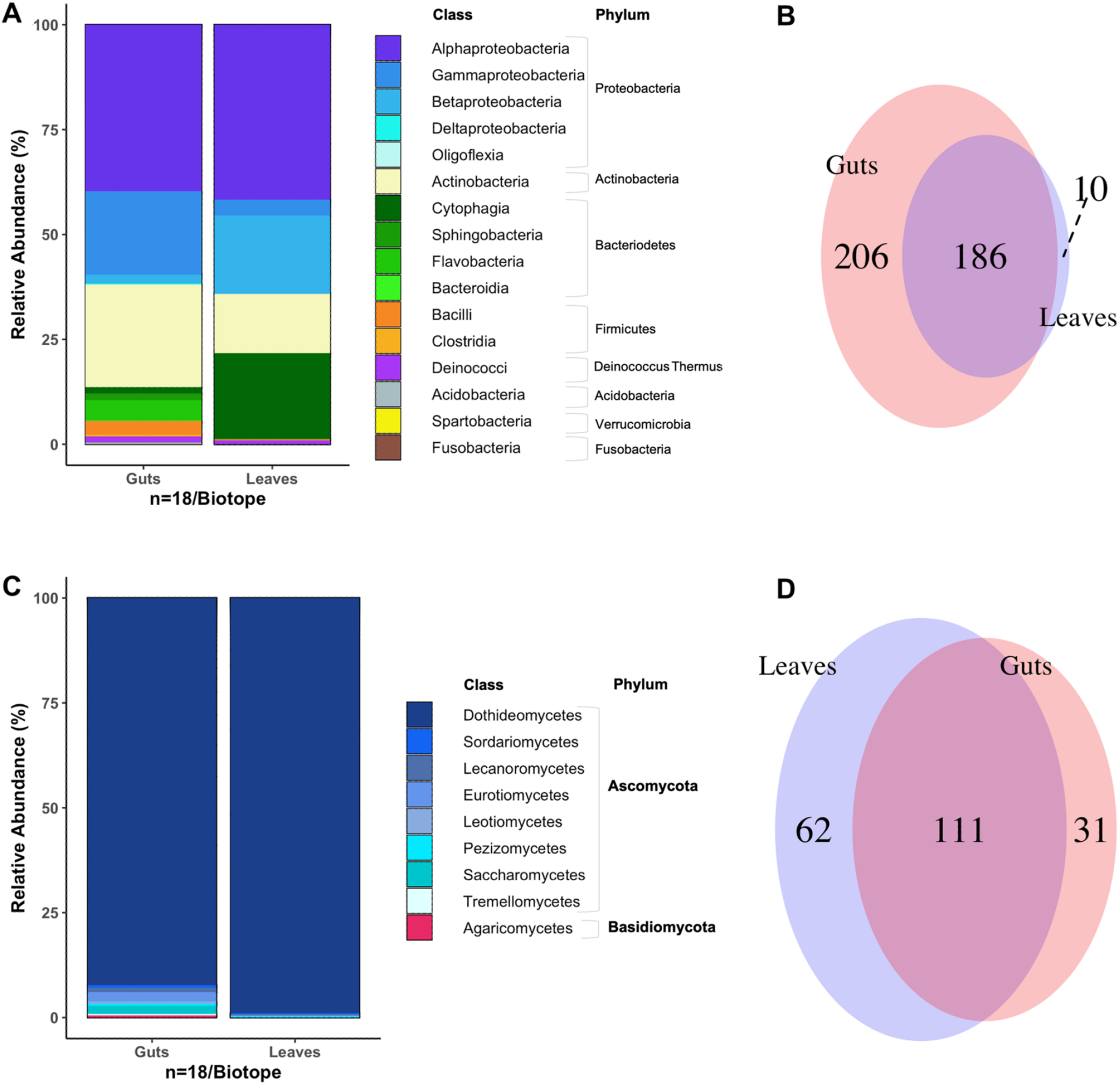
Taxonomic profiles of bacterial (**A**) and fungal (**C**) communities associated with the adult EAB gut and those associated with the leaves of the host trees. The taxonomic profile is based on the presence absence of ASVs in each biotope. The right part of the figure shows the number of ASVs shared and unshared between the two habitats for bacteria (**B**) and fungi (**D**).

On the 18 selected trees, the number of EABs collected per tree ranged from two to 100 adults (Fig. 1). According to the principal component analysis (PCA) analysis, the most contributing phytochemicals to the variability of the sampling sites were cellulose, acid fibre, sucrose, and total non-structural carbohydrate, (Supp. Fig. S1). Proteobacteria, Bacteriodetes, and Actinobacteria dominated the bacterial communities of the phyllosphere and EAB gut. A total of 186 amplicon sequence variants (ASVs) were shared by the two biotopes, 206 ASVs solely detected in the guts and 10 ASVs solely found in the leaves (Fig. 2A,B). Ascomycota was the main representative of fungal communities in the phyllosphere and the gut. The distribution of 111 ASVs encompassed both biotopes, whereas 31 ASVs were solely detected in the EAB gut and 62 ASVs in the leaves (Fig. 2C,D).

**Figure 3:**
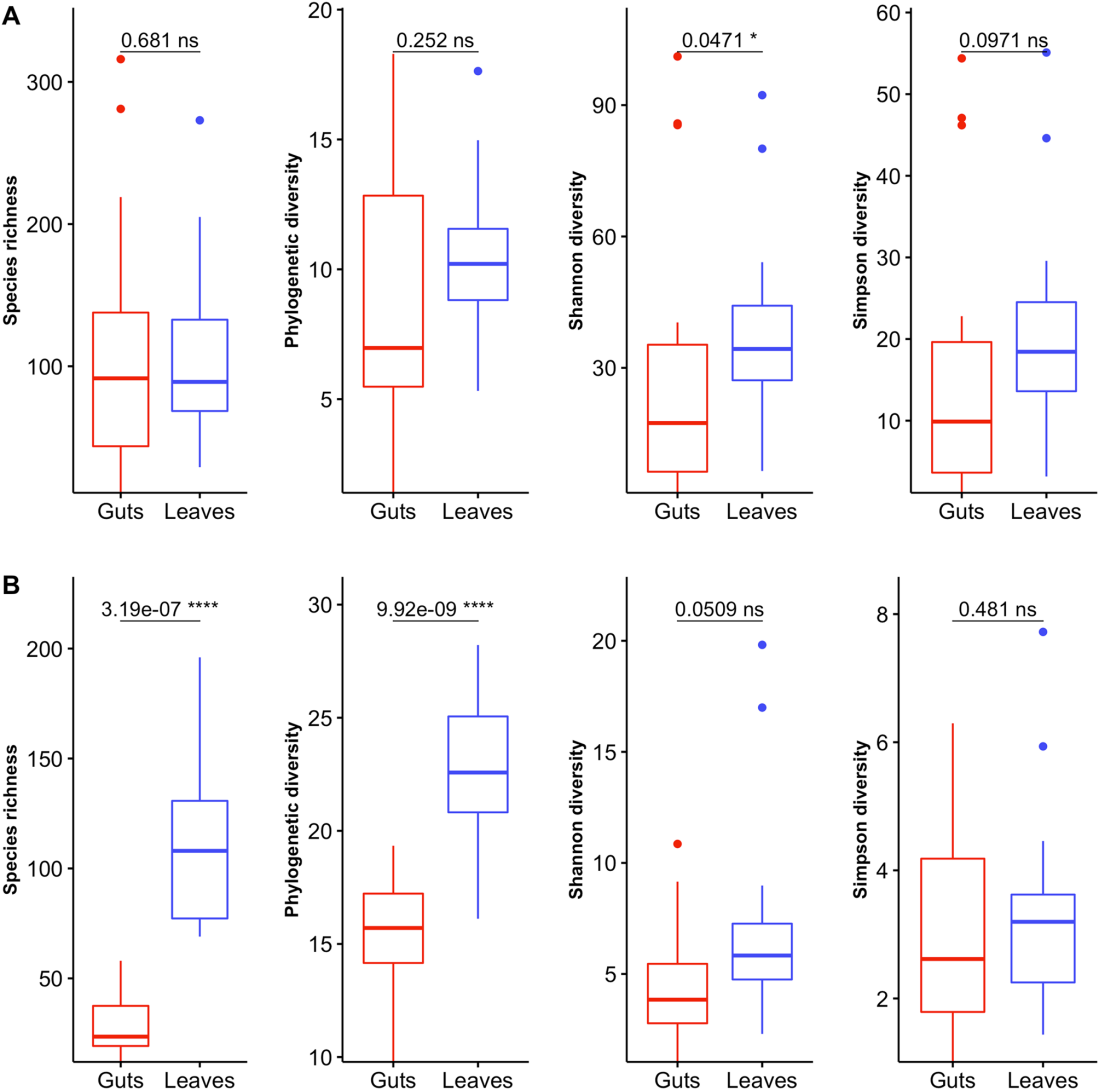
Species richness and diversity indices in the insect gut and on the leaves computed from raw data for communities of bacteria (**A**) and fungi (**B**). Values at the top of the panels: Wilcoxon signed-rank test statistics and significance: Significance codes: 0 ‘***’ 0.001 ‘**’ 0.01 ‘*’ 0.05 ‘NS’ > 0.05.

Alpha- and beta-diversity of leaves and guts microbiomes were compared and related to ash leaf phytochemicals to examine linkages between foliar biotic and abiotic features and the EAB microbiome. The species richness of the guts bacterial community was similar to that in leaves, with means of 109 ± 87 and 108 ± 62, respectively. Targeted α-diversity indices showed contrasting patterns in the two biotopes. There was no significant difference in diversity between the two biotopes for the Simpson diversity (guts: 0.83 ± 0.21, leaves: 0.93 ± 0.07) and for phylogenetic diversity (guts: 8.80 ± 4.82, leaves: 10.61 ± 3.01), whereas a lower Shannon diversity index was observed in the guts (2.82 ± 1.18) compared to leaves (3.53 ± 0.57) (Fig. 3A). The guts environment exerted a stronger filtering effect on fungal communities with lower species richness (28 ± 14) than the leaves (110 ± 37) and a constrained phylogenetic diversity in the gut (15.6 ± 2.4) compared to the leaves (22.81 ± 3.16). These responses were mostly driven by rare taxa because neither the Shannon diversity index (guts: 1.35 ± 0.65, leaves: 1.78 ± 0.56) nor the Simpson diversity index (guts: 0.56 ± 0.25, leaves: 0.63 ± 0.16) differed between the two biotopes (Fig. 3B). Alpha diversity parameters of the bacterial community associated with the guts were not related to phytochemical variables, but a significant relationship was found between fungal species richness in the guts and the cellulose content (F = 5.1629, *p* = 0.0382).

The EAB guts and ash leaves displayed contrasting bacterial (F = 8.94, adj. R^2^ = 0.18, *p* < 0.001) and fungal (F = 5.79, adj. R^2^ = 0.12, *p* < 0.001) community profiles, according to a db-RDA (Supp. Fig. S2). This dissimilarity between microbial profiles was supported by the occurrence of indicator ASVs (Table 1). Indicator ASVs for the guts were represented by 12 bacterial ASVs affiliated with Proteobacteria, while six bacteria (Proteobacteria and Bacteriodetes) and 14 fungi, mostly Dothideomycetes, were indicator ASVs for the leaves. Total variance in the microbial community between the gut and the leaves appeared higher for bacteria (0.77) than for fungi (0.55). Indeed, the bacterial community revealed more species (ASVs) with contributions to the β-diversity (SCBD) greater than the mean SCBD found across all the samples compared to the fungal community, with respectively 82 ASVs and 26 ASVs (data not shown). An overview of the local contribution to the β-diversity (LCBD) revealed greater values for the bacterial community in the insect gut (Supp. Fig. S3). More specifically, the bacterial community showed a higher LCBD mean value compared to the fungal community with respectively six sites (A06, A13, A14, A22, A28 and A32) and three sites (A18, A29, A35) having significant Holm-corrected LCBD *p* values (Fig. 1). Neither the LCBD nor the SCBD profiles were related to leaf phytochemicals based on the selection of explanatory variables in linear regression (data not shown). Similarly, the RDA constraining variation of the gut microbiome from the phytochemical profile was not significant. This decoupling between chemical and microbial profiles precluded consideration of phytochemicals in subsequent variation partitioning analyses.

**Table 1:** The indicator ASVs characterizing taxa strongly correlated to each biotope, identified using the indicator species analysis procedure implemented in the ‘indicspecies’ package.

ASVs^a^	Indval statistic	Phylum	Class	Order	Genus
**Adult EAB gut**					
Asvb11	0.913***	Bacteroidetes	Flavobacteria	Flavobacteriales	*Flavobacterium*
Asvb24	0.912***	Proteobacteria	Gammaproteobacteria	Enterobacteriales	*Serratia*
Asvb14	0.882***	Proteobacteria	Alphaproteobacteria	Caulobacterales	*Caulobacter*
Asvb28	0.881***	Proteobacteria	Alphaproteobacteria	Rhizobiales	*Bradyrhizobium*
Asvb47	0.878**	Proteobacteria	Gammaproteobacteria	Pseudomonadales	*Pseudomonas*
Asvb20	0.849***	Proteobacteria	Gammaproteobacteria	Alteromonadales	*Alishewanella*
Asvb63	0.816***	Proteobacteria	Gammaproteobacteria	Xanthomonadales	*Luteibacter*
Asvb31	0.782***	Proteobacteria	Gammaproteobacteria	Enterobacteriales	Unassigned
Asvb73	0.782***	Proteobacteria	Gammaproteobacteria	Enterobacteriales	Unassigned
Asvb25	0.782***	Proteobacteria	Alphaproteobacteria	Caulobacterales	Unassigned
Asvb64	0.745***	Proteobacteria	Gammaproteobacteria	Enterobacteriales	Unassigned
Asvb278	0.707***	Proteobacteria	Gammaproteobacteria	Pseudomonadales	*Pseudomonas*
**Ash leaves**					
Asvb874	0.999***	Bacteroidetes	Cytophagia	Cytophagales	*Hymenobacter*
Asvf1	0.957***	Ascomycota	Dothideomycetes	Pleosporales	Unassigned
Asvf39	0.942***	Ascomycota	Unassigned	Unassigned	Unassigned
Asvb776	0.913***	Bacteroidetes	Cytophagia	Cytophagales	*Hymenobacter*
Asvb625	0.913***	Bacteroidetes	Cytophagia	Cytophagales	*Hymenobacter*
Asvf3	0.913***	Ascomycota	Dothideomycetes	Pleosporales	Unassigned
Asvf11	0.890***	Ascomycota	Dothideomycetes	Pleosporales	Unassigned
Asvf23	0.875***	Ascomycota	Dothideomycetes	Myriangiales	Unassigned
Asvf7	0.862***	Ascomycota	Dothideomycetes	Pleosporales	*Pyrenochaeta*
Asvf48	0.849***	Ascomycota	Dothideomycetes	Dothideales	Unassigned
Asvb56	0.845***	Proteobacteria	Alphaproteobacteria	Sphingomonadales	*Sphingomonas*
Asvb68	0.843***	Proteobacteria	Alphaproteobacteria	Sphingomonadales	*Sphingomonas*
Asvf89	0.821***	Ascomycota	Dothideomycetes	Capnodiales	*Mycosphaerella*
Asvf86	0.816***	Ascomycota	Dothideomycetes	Dothideales	Unassigned
Asvf46	0.813***	Ascomycota	Dothideomycetes	Pleosporales	Unassigned
Asvf159	0.782***	Ascomycota	Dothideomycetes	Pleosporales	Unassigned
Asvf215	0.779***	Ascomycota	Dothideomycetes	Capnodiales	*Mycosphaerella*
Asvf346	0.751***	Ascomycota	Dothideomycetes	Pleosporales	*Lewia*
Asvb903	0.707***	Proteobacteria	Betaproteobacteria	Burkholderiales	*Massilia*
Asvf141	0.707***	Ascomycota	Unassigned	Unassigned	Unassigned

*** *p* ≤ 0.001; ** *p* ≤ 0.01.

The result was generated by using the multipatt() function of the indicspecies package, based on the species-site group association named Indval.g. The minimal significance alpha = 0.001 and 9999 permutations of samples among the two biotopes. The highest Indval statistic indicate the accuracy of the taxa and the maximum is 1.

^a^*Asvb* ASV assigned to bacteria, *Asvf* ASV related to fungi.

### Variation in gut microbes: partitioning of the observed variation

**Figure 4:**
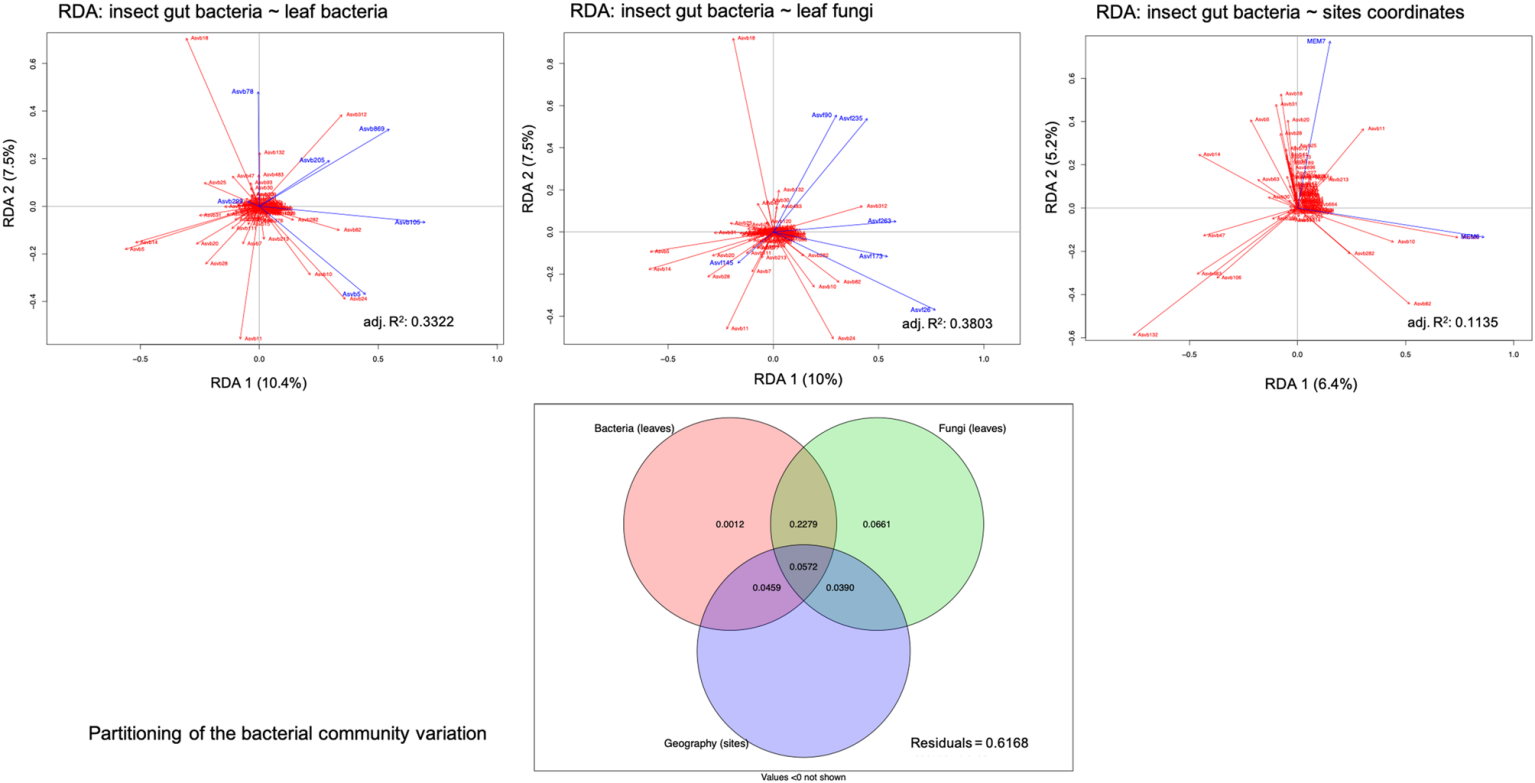
Variation partitioning of the gut bacterial community among three predictor matrices: bacteria associated with leaves (Hellinger-transformed), fungi associated with leaves (Hellinger-transformed), and dbMEM spatial eigenfunctions generated from the geographic coordinates of the sampling sites (*Fraxinus* trees). The selected explanatory variables (leaves) are represented in blue, and the response variables (insects’ gut) in red. Thus, the three explanatory matrices (bacteria, fungi, and geographic coordinates) are represented in individual RDA analyses. The adjusted R-square (adj.R^2^) corresponds to the R^2^ adjusted to the model containing all variables. The figure below the RDAs represents the partitioning variation analyses of the bacterial community associated with adult EAB gut.

The variation in the composition of the EAB gut microbiome was related to the leaf phyllosphere microbiome and the sampling site location. The variables that contributed the most to the changes in the EAB microbiome were selected in two partial RDAs**.** Six bacterial ASVs: ASVb105 (Gammaproteobacteria), ASVb869 (Gammaproteobacteria), ASVb78 (Actinobacteria), ASVb299 (Alphaproteobacteria), ASVb205 (Alphaproteobacteria), and ASVb5 (Actinobacteria), and six fungal ASVs from the Ascomycota, ASVf26, ASVf145, ASVf235, ASVf90, ASVf263, and ASVf173, associated with the leaves accounted for most of the variation in the composition of the gut bacterial community (Table 2). Two significant dbMEM, namely MEM6 (F = 2.12, adj. R^2^ = 0.06, *p* = 0.004) and MEM7 (F = 1.81, adj. R^2^ = 0.11, *p* = 0.048) were identified with the forward selection procedure followed by the RDA analysis performed on the geographical data. Selected variables explained 39.03% of the variation observed in the EAB gut bacterial community (Fig. 4). The three predictor matrices displayed 5.72% redundancy, whereas the shared contribution of phyllosphere bacteria and fungi showed the greatest effect on the gut bacterial community (22.79%). The six phyllosphere bacterial ASVs explained a modest 0.12% of the gut bacterial community structure, while the six leaf fungi ASVs explained 6.61% of the variation. The relationship between geographical site coordinates and the gut bacterial community was redundant, with the variation explained by the microbiome of the phyllosphere.

**Table 2:** Details on the selected ASVs of the variance of bacterial et fungal communities associated to adult EAB guts.

ASV^a^	Phylum	Class	Genus	Guts (n = 18) (%)	Leaves (n = 18) (%)
**Bacterial community**					
ASVb869	Proteobacteria	Gammaproteobacteria	Unassigned	16.7	33.33
ASVb78	Actinobacteria	Actinobacteria	*Kineococcus*	11.1	55.56
ASVb299	Proteobacteria	Alphaproteobacteria	Unassigned	16.7	33.33
ASVb205	Proteobacteria	Alphaproteobacteria	*Methylobacterium*	27.8	44.44
ASVb105	Proteobacteria	Gammaproteobacteria	*Acinetobacter*	16.7	44.44
ASVb5	Actinobacteria	Actinobacteria	*Propionibacterium*	88.9	100.00
Asvf26	Ascomycota	Unassigned	Unassigned	5.6	55.56
Asvf145	Ascomycota	Unassigned	Unassigned	0.0	44.44
Asvf235	Ascomycota	Leotiomycetes	*Naevala*	5.6	33.33
Asvf90	Ascomycota	Sordariomycetes	*Diaporthe*	0.0	38.89
Asvf263	Ascomycota	Dothideomycetes	Unassigned	5.6	33.33
Asvf173	Ascomycota	Dothideomycetes	Unassigned	0.0	38.89
**Fungal community**					
Asvb52	Actinobacteria	Actinobacteria	Unassigned	27.8	55.6
Asvb633	Proteobacteria	Alphaproteobacteria	Unassigned	5.6	50.0
Asvb10	Proteobacteria	Gammaproteobacteria	*Escherichia*/*Shigella*	55.6	44.4
Asvb740	Proteobacteria	Betaproteobacteria	*Massilia*	0.0	38.9
Asvb896	Proteobacteria	Alphaproteobacteria	*Sphingomonas*	38.9	66.7
Asvb114	Actinobacteria	Actinobacteria	Unassigned	22.2	50.0
Asvb503	Proteobacteria	Alphaproteobacteria	*Methylobacterium*	27.8	66.7
Asvf486	Ascomycota	Dothideomycetes	*Phaeosphaeria*	5.6	55.6
Asvf292	Ascomycota	Dothideomycetes	Unassigned	0.0	44.4
Asvf8	Ascomycota	Dothideomycetes	*Phoma*	61.1	100.0
Asvf123	Ascomycota	Dothideomycetes	Unassigned	11.1	55.6

In both cases, the values correspond to the percentage of presence of the ASV taxa in all the samples. Only the ASV found to be indicators of a host are shown.

^a^*Asvb* ASV assigned to bacteria, *Asvf* ASV related to fungi.

**Figure 5:**
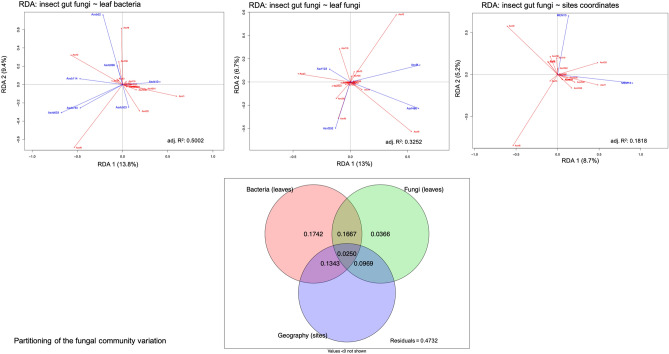
Variation partitioning of the gut fungal community among three predictor matrices: bacteria associated with leaves (Hellinger-transformed), fungi associated with leaves (Hellinger-transformed), and dbMEM spatial eigenfunctions generated from the geographic coordinates of the sampling sites (*Fraxinus* trees). The selected explanatory variables (leaves) are represented in blue, and the response variables (insects’ gut) in red. Thus, the three explanatory matrices (bacteria, fungi, and geographic coordinates) are represented in individual RDA analyses. The adjusted R-square (adj.R^2^) corresponds to the R^2^ adjusted to the model containing all variables. The figure below the RDAs represents the partitioning variation analyses of the fungal community associated with adult EAB gut.

**Figure 6:**
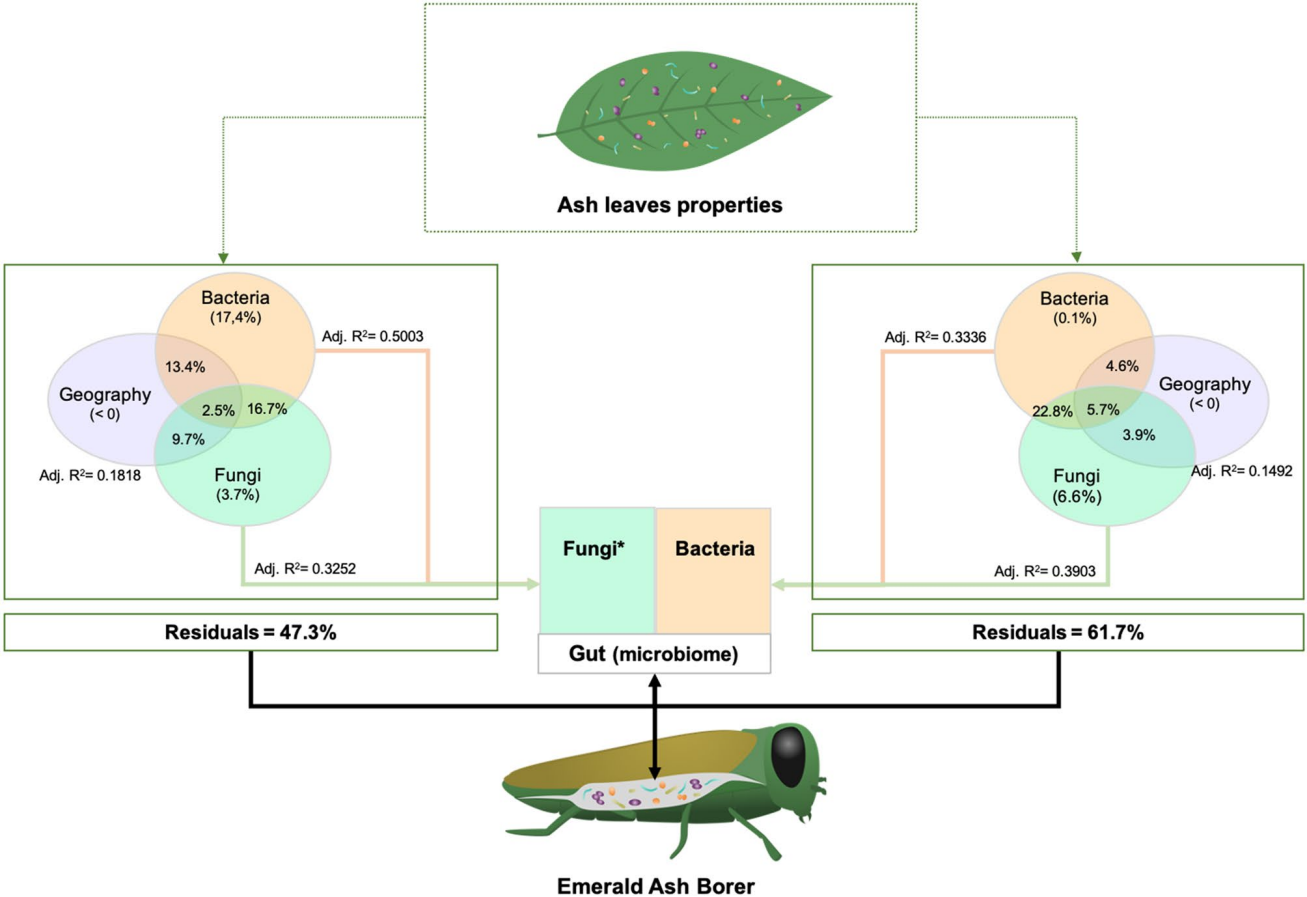
Global representation showing variation partitioning analysis of microbial communities associated with leaves on the microbial communities associated with adult EAB gut. The host tree descriptors that have been found as explanatory matrices (bacteria, fungi, and geographic coordinates) are represented on the left portion (for the variation of the EAB gut fungal community), and on the right portion (for the variation of the EAB gut bacterial community). The dash box indicates the explanatory variable (properties of the host tree), and the values indicated in the circle correspond to the percentage of explanation. *After computing a forward selection (alpha < 0.05) prior to multiple regressions. The cellulose content significantly explained the species richness observed in the fungal community associated with the adult EAB gut.

Seven bacterial ASVs, ASVb52 (Actinobacteria), ASVb633 (Alphaproteobacteria), ASVb10 (Gammaproteobacteria), ASVb740 (Betaproteobacteria), ASVb896 (Alphaproteobacteria), ASVb114 (Actinobacteria), and ASVb503 (Alphaproteobacteria), and four fungal ASVs, ASVf486, ASVf292, ASVf8, and ASVf123, all associated with the phyllosphere and belonging to the Dothideomycetes, explained the variation in the gut fungi. The forward selection procedure followed by the RDA analysis performed on the geographical data identified MEM14 (F = 3.2058, adj. R^2^ = 0.12, *p* = 0.001), and MEM13 (F = 2.11, adj. R^2^ = 0.18, *p* = 0.031) as significant variables explaining the variation in gut fungi. The phyllosphere microbiome and the sampling site location explained 52.68% of the variation observed in the gut fungal community (Fig. 5). The individual contribution of the phyllosphere bacterial community to the variation in the EAB gut microbiome was higher on the fungal community than on the bacterial community, with respectively 17.42% and 0.12%. The individual contribution of the geographic coordinates was not significant (< 0%), while its common contribution to the bacteria and fungi was 13.43% and 9.69%, respectively. The common contribution of the three predictor matrices was 2.50%. The results indicate a stronger relationship between the ash phyllosphere microbiome and the gut fungi than the gut bacteria (Fig. 6 and Table S2).

## Discussion

The composition of the insect gut microbiome is influenced by diverse environmental drivers including developmental stage, diet, season, and plant defences. The role of diet in determining gut microbiome structure has been mostly related to the host’s nutritional requirements for carbohydrates, vitamins, amino acids, and inorganic elements^29^. These compounds are supplied either directly by leaves (carbohydrates and inorganic nutrients) or indirectly through microbial metabolism of leaf residues (amino acids, vitamins, and inorganic nutrients)^29^. Microbiome studies are expected to shed light on the interplay between the microbiome of the insect gut and host fitness, and therefore can increase basic knowledge of insect biology for the design of novel control methods. One limitation on this quest is the multiple filtering effects exerted on insect gut microbiome by abiotic and biotic influences. In the case of diet, it is reasonable to expect food to have both abiotic and biotic effects on the gut microbiome structure, but the impact of food-associated fungi and bacteria has not been documented. DNA sequencing approaches covering the overall structure of microbial communities, including living cells and relic DNA from dead cells or debris^30^ were proposed to bridge this knowledge gap. Although the distribution and relative abundance of living cells has been directly linked to host fitness, relic DNA is a legacy from the environment, and Lennon, et al.^31^ maintained that relic DNA might be one of the largest pools of nucleic acids in the biosphere. In this study, disentangling the contribution of such a legacy on the host microbiome structure was made possible by selecting EAB adults known to be *Fraxinus spp*. host specialists. Both insect and host tree were examined to evaluate the covariation between host tree biotic and abiotic features and insect gut microbiome structure.

Dissimilarities between ash leaf and EAB gut biotopes were reflected in variations in the bacterial and fungal communities and indicator ASV levels (Fig. 2 and Table 1). The phyllosphere is exposed to solar radiation, pollution, and insect herbivory^27,32–36^, while most insect gut environments are alkaline and protected from environmental influences except through feeding. In spite of these contrasting conditions, the microbiomes of both biotopes exhibited similar diversity according to the computed diversity indices. However, the significantly lower phylogenetic diversity in the gut fungal community, brought up the question whether the phylogenetic diversity could be related to the ecological process happening in related the fungal community of each biotope. Fungi and insects have been largely documented to have mutualistic relationships, such as in those insect species that feed on wood or woody detritus. The fungal activities that benefit insects include the breakdown of cellulose or other recalcitrant molecules and protection through synthesis of repellents and antimicrobial metabolites^37^.

Interestingly, the ash leaf cellulose content was related to the fungal community associated with the adult EAB gut. Although some insects can synthesize their own cellulases, the enzymatic activity of symbiotic microorganisms is often needed for complete cellulolysis^29,38^. Some microorganisms, including fungi, have been shown to possess enzymes capable of hydrolysing a wide variety of plant materials^39^. Mittapalli, et al.^40^ even found microbial transcripts coding for cell-wall degrading enzymes in the transcriptome of the EAB larvae midgut. The variation partitioning approach highlighted the covariations of bacterial and fungal profiles associated with both biotopes.

Compelling evidence supports the idea that environmental microorganisms play a major role in shaping the insect gut microbiome. For instance, gut colonization of a lepidopteran species (*Pieris brassicae*) by the bacterium, *Pseudomonas protegens*, commonly associated with plants and soil is promoted by the disruption of the commensal microbiome in the insect gut induced by the bacterium^41^. The potential effects of food-associated microbes on gut microbiome has been documented in some fish species, confirming the role of food associated microbes in the prediction of population differences in the gut microbiota^42^. To the best of our knowledge, there has been no previous report on the effects of phyllosphere microbiome on the gut microbial community of a herbivorous insect. Here, the fungal and bacterial communities associated with the ash phyllosphere explained 53 and 48% of the variation observed in the adult EAB gut microbiome. We found that the gut bacterial community might be less sensitive to the phyllosphere microbiome than the gut fungal community. A previous work by Zhang et al.^5^ investigating the bacterial and fungal communities in the gut of adult *Agrilus mali* ingesting different diets, revealed a greater change in the fungal community than in the bacterial. Based on our results, we would suggest that the changes in the fungal community associated with the *Agrilus mali* gut could be due to food-associated microorganisms. Both environmental fungi as a complement of insect diet and dissemination of gut-associated fungi in the environment can explain the covariation between the EAB gut and the phyllosphere microbiomes. However, more investigation is needed to better understand the potential relationships between insect gut fungi and the fungi associated with the phyllosphere of food plants, as well as the influence of gut bacteria on insect metabolism. Variation partitioning analysis showed significant covariation of the bacterial and fungal communities associated with the ash phyllosphere and the geographic coordinates of the host sites with the EAB gut microbiome. In each variation partitioning analysis, the joint redundancy of the phyllosphere bacterial and fungal communities was higher than the unique proportions explained by bacterial and fungal communities (Fig. 5). High redundancy suggests biological interactions between bacteria and fungi occurring in both leaf and gut biotopes^43^. Greater knowledge about the mechanism of microbial interactions may improve our understanding of the biological interactions among coexisting microbial communities.

The successful adaptation of insects to environmental changes has been reported in many papers. According to Gupta and Nair^44^, microbes may have played a crucial role in insect survival, and gut bacteria may facilitate faster adaptations of an insect host to a changing environment. Schowalter^29^ also reported that insect responses to environmental conditions determined their survival, reproduction, and fitness. Variation in the microbial composition in the EAB gut is certainly related to their adaptation to the host tree properties. As to our results demonstrating the covariation of the phyllosphere-host microbiomes, future investigations are needed to elucidate the mechanism behind the microbiome variation and, more importantly, the consequences of that variability on the adaptation of insects facing environmental changes. Understanding how beneficial functional traits can be provided to EAB through ingestion of microbiota associated with ash leaves could provide valuable insights into the contribution of environmental microorganisms to insect fitness, and how this might be manipulated to human advantage in the case of pest species such as the EAB.

## Materials and methods

### Sampling site locations, insect collection, and leaf sampling

#### Site locations

Eighteen white ash trees, *Fraxinus americana* L., separated by a radius of at least 200 m from any other ash tree, were randomly selected in the Lanaudière region North-East of Montréal, QC, Canada (Fig. 1). White ash trees were identified by CG using a tree identification key^45^.

#### Sampling of adult EABs

Adult EABs were collected using 12-funnel green Lindgren traps (Synergy Semiochemicals Corp., Burnaby, BC, Canada) deployed in the highest third of the canopy of each selected ash tree using a catapult (North Big Shot, Sherriltree, Greensboro, NC, USA) to set up a permanent halyard system for regular trap monitoring. Based on previous observations on adult flight activity and the calculation method of degree-days^11^, traps were deployed and monitored after a period of 341.7 degree-days (base 10 °C) was reached. Adults were collected twice weekly in 2018, from June 21st to August 3rd. For each selected tree and each sampling date, insects were transferred into a disinfected plastic container using sterilized tweezers and supplied with fresh ash tree leaves as substrate and food source. Containers were kept on ice during transport to the laboratory. Each healthy insect was immediately transferred into labelled sterile 1.5 mL microfuge tubes, frozen at − 80 °C, and stored until DNA extraction. The number of insects collected per tree ranged from two to 100 EAB adults.

#### Leaf sampling

For each selected tree, for each selected tree, several leaves surrounding the trap were collected using a 10-m aluminium extendable pole tree pruner. Separate samples were placed in sterile bags and transported on ice to the laboratory at the fourth sampling week. Subsamples of each tree corresponding to five leaves randomly selected were then stored at − 80 °C before DNA and metabolite extractions. Until digitalization, foliar voucher samples are available to Guertin’s laboratory for further consultation.

Several site locations were visited to collect leaf samples and deployed traps to catch insects. For each place where plants and EAB adults have been collected, we obtain the authorization of private landowners and municipalities before collecting and using them in this study.

Our study complies with relevant institutional, national, and international guidelines and legislation.

### DNA extraction

For each tree, insect DNA extraction was performed on two randomly selected EAB adults. The wings were first removed with sterile tweezers and scissors, and the exoskeleton was sterilized by agitating (Fisher Vortex Genie 2, Ottawa, ON, Canada) the beetle in 1 mL of 70% ethanol for 1 min. Beetles were rinsed with 1 mL of sterile water by vortexing for 30 s. Dissection was performed in sterile phosphate-buffered saline, the two guts were pooled, and total genomic DNA was extracted by the mechanical lysis as described by Mogouong, et al.^11^. For each leaf sample, endophytes and epiphytes were processed together by grinding five leaflets corresponding to the apical leaflet of five leaves randomly selected (Supp. Fig. S4) in liquid nitrogen and using the MoBio PowerSoil DNA isolation kit (Qiagen, Toronto, ON, Canada). DNA extracts from insect guts and leaves were purified using a PowerClean Pro DNA clean-up kit (Qiagen, Venlo, the Netherlands). DNA concentration was estimated using the Quant-iT PicoGreen dsDNA assay kit (Invitrogen, Life Technologies, Burlington, ON, Canada) following the manufacturer’s instructions.

### DNA amplification by PCR, amplicon sequencing and data processing

The v6-v8 region of the 16S rRNA gene of bacteria and the ITS2 region for fungi were sequenced using, respectively, the primers B969F-CS1 (5′-ACGCGHNRAACCTTACC-3′) and BA1406R-CS2 (5′-ACGGGCRGTGWGTRCAA-3′)^46^ and the primers ITS3_KYO2 (5′-GATGAAGAACGYAGYRAA-3′) and ITS4_KYO3 (5′-CTBTTVCCKCTTCACTCG-3′)^47^ for both insect and leaf DNAs. Sequencing was done using an Illumina Miseq with 2 × 250 bp paired-ends at the Quebec Genome Innovation Centre (Sainte-Justine Hospital, Montreal, QC, Canada). Sequence data were processed with USEARCH, v10.0.240^48,49^, using a pipeline for constructing amplicon sequence variants (ASVs)^50,51^. The ASV matrix returned frequencies of ASVs per sample. The main steps of the pipeline included quality filtering, dereplication by finding unique sequences, merging of paired-end reads (total length of 438–484 bp, for bacteria, or 379–450 bp, for fungi), clustering of ASVs, creation of the ASVs abundance table (an ASV was generated with at least eight sequences with 100% identity) after removing the chimeras, and lastly taxonomy assignment. The taxonomy assignment was completed using the RDP (Ribosomal Database Project)^52,53^ v.16 training set of 16S rRNA genes for bacteria and the RDP Warcup training set v.2 for fungi. Only assignments > 80% were considered. The non-assigned ASVs were identified by the label, ‘non-assigned’. The bacterial ASV table comprised 1073 ASVs represented by 326,583 sequences, while the fungal ASV table comprised 602 ASVs clustered in 1,431,817 sequences.

### Extraction of leaf phytochemicals

#### Total soluble proteins

Extraction of total soluble protein was performed as described by Chen and Poland^54^ and Bi et al.^55^. Briefly, 40 mg of ash leaves were ground in liquid nitrogen and transferred to tubes containing 5 mL of 0.1 M ice-cold phosphate buffer (pH 7.0) containing 1% polyvinylpolypyrrolidone. After vortexing and centrifugation at 10,000 × g for 10 min at − 2 °C, the total protein content in the supernatants was determined using the bicinchoninic acid (BCA) protein assay. The absorbances of the reaction mixture were read at 562 nm using a Tecan Infinite M1000 Pro microplate reader (Tecan US, Morrisville, NC, USA). The protein concentrations were calculated from a standard curve of bovine serum albumin (BSA) and expressed as µg of protein per g of dry leaf mass (µg/g DW) after subtracting the water content of each sample.

#### Total phenolics

Total phenolic content was determined as described by Torti et al.^56^ and Hagerman^57^. After removing prominent veins, fresh leaves were ground, and 200 mg was extracted in 5 mL of 70% acetone. The samples were sonicated for 30 min at 4 °C and then centrifuged at 16,000 × g for 15 min at 4 °C. The amount of extract corresponding to the pooling of three successive extractions was used for the analysis. The total phenolic content was determined using the Folin-Ciocalteu reagent assay described by Beauchemin et al.^58^ with some modifications. First, 50 µL of each extract was mixed with 2.5 mL of Folin-Ciocalteu reagent and incubated in a 40 °C water bath for 8 min. Then, 1 mL of sodium carbonate 1 M was added and samples were incubated at 40 °C for one hour in the dark. Aliquots of each sample’s reaction products were transferred to a 96-well microplate and absorbance was measured at 765 nm using a SpectroStar Nano spectrophotometer (BMG Labtech, Germany) with gallic acid standards to determine the total phenolic content expressed in mg of gallic acid equivalents per g of dry leaf mass, based on the water content of each sample (mg/g DW).

#### Chlorophyll and carotenoids

Using the dimethyl sulfoxide (DMSO) method described by Garg^59^, the chlorophyll and carotenoid content were determined. Briefly, 50 mg of fresh leaf tissue, cut in pieces of approximatively 1 × 1 cm, were placed in a tube containing 7 mL DMSO, and incubated at 65 °C for 3 h. After removing the leaves, the extracts were transferred to graduated tubes, and the volume was adjusted to 10 mL with DMSO. Absorbance was measured at 480 (A_480_), 645 (A_645_), and 665 nm (A_665_) using a HACH DR2800 spectrophotometer (Hach Canada, London, ON, Canada), and chlorophyll and carotenoid concentrations were calculated based on the following formulas from Wellburn^60^:

$${\text{Chlorophyll}}\;{\text{a}}\;\left( {\upmu {\text{g}}\;{\text{mL}}^{ - 1} } \right) = 12.19*{\text{A}}_{665} {-}3.45*{\text{A}}_{645}$$

$${\text{Chlorophyll}}\;{\text{b}}\;\left( {\upmu {\text{g}}\;{\text{mL}}^{ - 1} } \right) = 21.99*{\text{A}}_{645} {-}5.32*{\text{A}}_{665}$$

$${\text{Carotenoids}}\;\left( {\upmu {\text{g}}\;{\text{mL}}^{ - 1} } \right) = \left( {1000*{\text{A}}_{480} {-} \, 2.86*{\text{chlorophyll}}\;{\text{a}}{-}129.9*{\text{chlorophyll}}\;{\text{b}}} \right)/221.$$

The final concentrations were expressed as mmol per g of dry mass (mmol/g DM).

#### Soluble carbohydrates and starch

Soluble carbohydrates and starch contents of leaves were determined as described by Marquis et al.^61^. For each sample, fresh leaves were ground in liquid N_2_ and 30 mg was placed in a centrifuge tube containing 1.5 mL ethanol 80% (v/v) and incubated in a water-bath at 80 °C for 8 min. Following centrifugation (10,000 × g at 2 °C) for 10 min, the supernatants were collected (extract A), and the extraction procedure repeated one time (extract B). The soluble carbohydrates were pooled (extracts A and B) for quantification. The pellets containing insoluble starch were dehydrated overnight at 50 °C. The tube containing the pellet was rinsed with 80% ethanol to remove any soluble carbohydrates. The starch was hydrolysed by addition of 2.5 mL of acetate buffer (0.2 M, pH 4.5) to the pellets and incubation in boiling water for one hour. After cooling to room temperature, 2 mL of acetate buffer and 1 mL of glucoamylase (20 units/mg at pH 6.0) at 0.5% (Bio Basic Inc., ON, Canada) were added to the mixture, incubated at 55 °C for 8 h, and supernatants were collected (extract C). The soluble carbohydrate extracts (A and B pooled) and the starch hydrolysate (extract C) were then filtered through 0.45 µm syringe filters (Bio Basic Inc. ON, Canada). Soluble sugars were quantified and analysed using a high-performance liquid chromatograph (HPLC, Shimadzu) equipped with a pump (LC-10VP), an autosampler (SIL-10AXL), a column oven (CTO-10ASVP) at 65 °C, a refractive index detector (RID-10A), a system controller (SCL-10AVP), and an Aminex HPX-87H column (300 mm, 7.8 mm). The mobile phase consisted of 5 mM H_2_SO_4_ at 0.6 ml min^−1^. The soluble carbohydrate extract concentration was determined by quantifying specific sugars (sucrose, glucose, fructose) against their respective standards (D-sucrose, D-glucose, D-fructose). Starch concentration was measured by quantifying hydrolysed products (glucose) against the glucose standard and expressed in glucose equivalents. Finally, the non-structural carbohydrate (TNC) content was computed by combining the content of the three extracted sugars (glucose, fructose, and sucrose) and the hydrolysed starch (D-glucose equivalents). The sugar fraction was expressed in mg per g of dry mass (mg/g DM).

#### C and N content of leaves

Leaves were dried at 70 °C for 72 h and ground. The carbon and nitrogen content, and the carbon-to-nitrogen ratio were determined by combustion using an Elementar Vario Micro Cube (Elementar, Germany) and acetanilide as the standard. The amount of carbon and nitrogen were expressed as percentages.

#### Leaf degradability

The degradability of leaf fibre was estimated by extracting tissue fractions as described by García et al.^62^ and using an ANKOM fibre analyzer (Ankon Technology, Macedon, NY, USA). The fractions extracted, including neutral detergent fibre (NDF), acid detergent fibre (ADF), acid detergent lignin (ADL), cellulose and hemicellulose, were expressed as percent of tissue^62,63^.

### Statistical analyses

#### Richness and diversity

The raw data was used to assess the α-diversity expressed as species richness and ecological diversity indices. The species richness and diversity were investigated by computing the species richness and two diversity indices (Shannon and Simpson, with corresponding Hill numbers) using the diversity() function available in the ‘vegan version 2.5-6’ package^64^ and following the procedure proposed by Borcard et al.^65^. Faith’s phylogenetic diversity (PD) calculation allowed us to determine the environmental diversity patterns^66^. The PD, corresponding to the sum of the branch lengths belonging to the minimum spanning path, was computed based on cladistic information^67^ using the PD() function available in the ‘picante version 1.8.1’ package^68^. The computed diversity indices of microbial communities associated with the insect guts and the leaves were compared using Wilcoxon tests.

#### Microbiome composition variation

Among sites, the β-diversity defined as the variation in community composition among sampling sites (i.e., the 18 sampled trees) was investigated for the microbial communities in the adult EAB guts’ and on the leaves, without reference to an explicit gradient. The β-diversity was determined by three complementary approaches after removing ASVs whose frequency in the ASV table was < 0.005% and performing the Hellinger transformation. The first approach, proposed by Legendre and De Cáceres^69^, calculates the local contributions of each site to β-diversity (LCBD), defined as “comparative indicators of the uniqueness of a site” and based on the ASVs’ composition and the species contributions to the β-diversity (SCBD), which represents the level of contribution of each ASV to the overall β-diversity. LCBD and SCBD were computed using Hellinger-transformed ASV frequencies with the beta.div() function available in the ‘adespatial version 0.3-8’ package^70^. The final values of SCBD and LCBD, ranging from 0 to 1, indicated the importance of the species and the sites in the overall β-diversity for each microbial community. A prerequisite of the method is the evaluation of the total variance of the Hellinger-transformed ASV matrix, called BD_Total_, which represents an estimate of the β-diversity.

BD_Total_ = SS_Total_/(n − 1), where SS_Total_ corresponds to the total sum of squares for Hellinger-transformed ASV abundances, and n is the total number of sites. BD_Total_ was then decomposed into LCBD (relative contribution of the site i to the β-diversity) and SCBD (relative contribution of ASV column j to the β-diversity) indices computed as follows:

LCBD = SS_i_/SS_Total_, where SS_i_ corresponds to the contribution of the site i to the overall β-diversity.

SCBD = SS_j_/SS_Total_ where SS_j_ corresponds to the contribution of ASV column j to the overall β-diversity.

The second approach examines the distance between the two biotopes (adult EAB guts and ash leaves) based on the ASV frequencies of the microbial members of the two groups. We performed a distance-based redundancy analysis (db-RDA with Bray–Curtis distance) by using the capscale() function of the ‘vegan’ version 2.5-6 package^64^. Briefly, a Bray–Curtis distance matrix of samples from adult EAB guts and the phyllosphere was constructed and used to determine the principal coordinates that were then used to analyse its relationship with each biotope using RDA. The model’s significance was validated by a permutation test using the function anova.cca() of the ‘vegan’ version 2.5-6 package. The third β-diversity analysis was based on the concept of indicator species proposed by De Cáceres, et al.^71^ identifying ASV indicators that could reflect the state of the environment using the multipatt() function available in the ‘indicspecies’ version 1.7.9 package^72^.

#### Relationships between ash leaf phytochemicals and the microbiome in the adult EAB gut

Relationships between the extracted phytochemicals and the diversity indices computed for the microbial communities associated with the adult EAB gut were analysed using the permutational selection approach proposed by Blanchet, et al.^73^ based on the forward selection of explanatory variables in a linear regression function available in the ‘vegan’ package. Briefly, the forward.sel() function of the ‘adespatial’ package selects the best explanatory variable(s) in an explanatory matrix (phytochemical dataset) which suits a predefined model (ASV frequencies) with a preselected significant p value (0.05). Selected molecules were used in a regression model and tested with a permutation test using the function anova.cca() available in the ‘vegan’ package. The relationship between the computed LCBD indices and the phytochemicals were investigated using the same approach.

#### Partitioning of the variation

**Figure 7:**
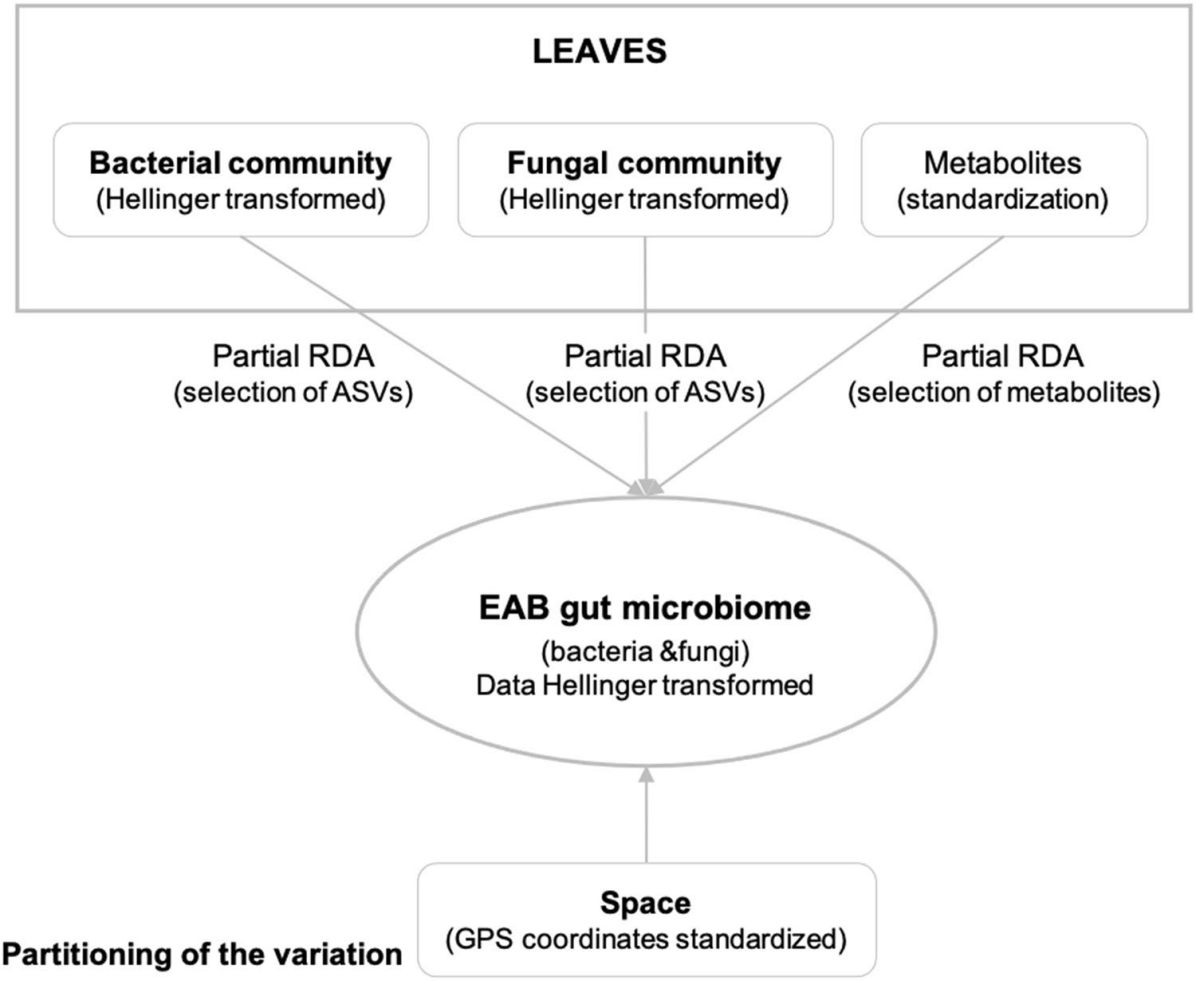
Step-by-step representation of the two ‘variance partitioning’ analyses, including the variables datasets concerned. The circles show the response dataset, and each rectangle corresponds to a dataset of predictor variables. The response variables were transformed before the variance partitioning analysis.

Relationships between the microbial communities related to the adult EAB guts and their food source, represented by ash leaf phytochemicals and associated microbial communities, were analysed following the approach proposed by Borcard, et al.^13^ and Peres-Neto, et al.^14^. Briefly, data matrices of predictors related to the ash leaves (bacterial community, fungal community, phytochemicals, and geographic positions of sample sites (18 sampled ash trees) were tested as predictor variables of the bacterial and fungal communities in EAB guts. The ASV matrices utilized for variation partitioning contained a subset of ASVs whose occurrence was observed in at least 30% of the samples. This arbitrary cutoff was selected to eliminate rare ASVs with sparse distribution profiles. Thus, each predictor matrix was used in a partial RDA after a preliminary Hellinger transformation of the ASV abundance matrices. The sampling sites’ geographic coordinates were included as a potential predictor matrix after transforming them into distance-based Moran’s eigenvector maps (dbMEMs). The forward.sel() function was used to select the significant dbMEMs before the test of significance in a partial RDA and the variance partitioning analysis. Lastly, the variation partitioning of the microbial communities in the adult EAB gut enabled us to disentangle the contribution of each evaluated matrix. The variation partitioning was separately performed for the bacterial and fungal communities associated with the adult EAB gut by using the varpart() function of the ‘vegan’ package and following the procedure of Borcard, et al.^65^ (Fig. 7).

## Supplementary Information

Supplementary Information.

## Data Availability

Raw data is available in the Sequence Read Archive of the National Centre for Biotechnology Information (SRA, NCBI) under the BioProject Number (PRJNA645095) and the SRA (SRP271139).
