# Supplementary material for: The phyllosphere microbiome of host trees contributes more than leaf phytochemicals to variation in the Agrilus planipennis Fairmaire gut microbiome structure
Source: Sci Rep. 2021 Aug 5;11:15911. doi: 10.1038/s41598-021-95146-9 (PMC8342481; doi:10.1038/s41598-021-95146-9)
Supplement: Supplementary file 1 — Supplementary Information. [file 41598_2021_95146_MOESM1_ESM.pdf]

The phyllosphere microbiome of host trees contributes more than leaf phytochemicals to variation in the *Agrilus planipennis* Fairmaire gut microbiome structure

Authors

Judith Mogouong[1], Philippe Constant[1], Pierre Legendre[2], and Claude Guertin[1]

Institutional address

[1]Institut National de la Recherche Scientifique, Centre Armand-Frappier Santé Biotechnologie, Laval, QC, Canada, H7V 1B7

[2]Département de sciences biologiques, Université de Montréal, C.P. 6128, succ. Centre-ville, Montréal, Québec H3C 3J7, Canada

E-Mail address

judith.mogouong@gmail.com

Pierre.Legendre@UMontreal.ca

philippe.constant@inrs.ca

claude.guertin@inrs.ca

Corresponding author

Claude Guertin

Keywords: *Agrilus planipennis* Fairmaire, ash leaf, cellulose, emerald ash borer, gut microbiome, *Fraxinus americana*, leaf microbiome, microbe-plants-insect interactions, microbial diversity, microbiome, phyllosphere, variation partitioning.

Supplementary materials

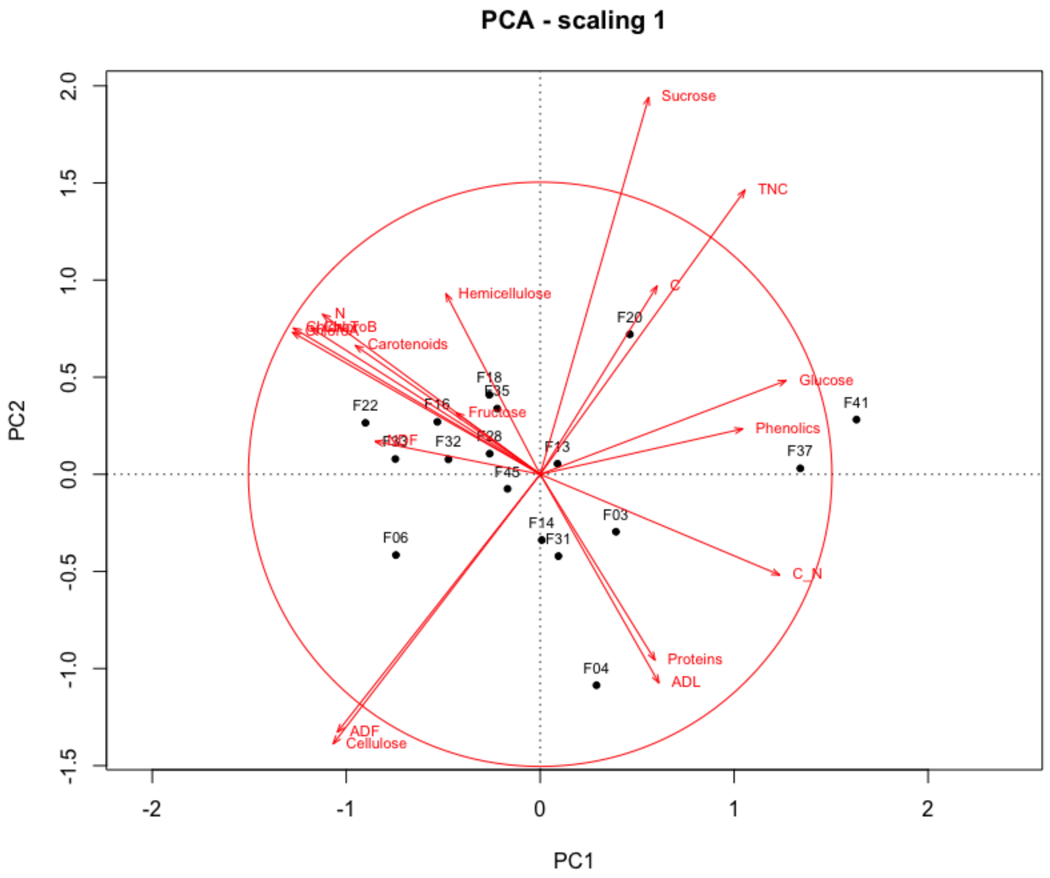

Supp. Fig. S1. The principal component analysis (PCA) performed with the eighteen phytochemicals extracted from ash leaves. The values were standardized and scaled before prior to the PCA analysis. Four molecules appeared as contributing the most to the dispersion of the sites in reduced space (two-dimensions): the cellulose, the acid detergent fibre fraction (ADF), the sucrose, and the total non-structural carbohydrates (TNC)

A B

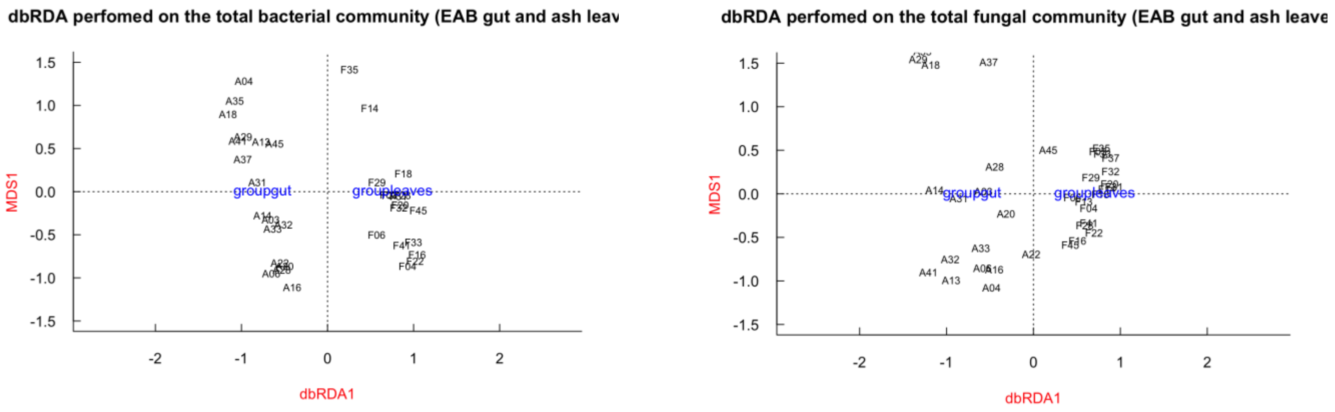

Bacteria

	Df	SumOfSqs	F	p(>F)
Model	1	1.3375	5.7853	0.001***
Residual	34	7.8605		

Fungi

	Df	SumOfSqs	F	p(>F)
Model	1	1.3375	5.7903	0.001***
Residual	34	7.8536		

Supp. Fig. S2. db-RDA analysis of bacterial (A) and fungal (B) communities associated to adult EAB gut and to leaves.

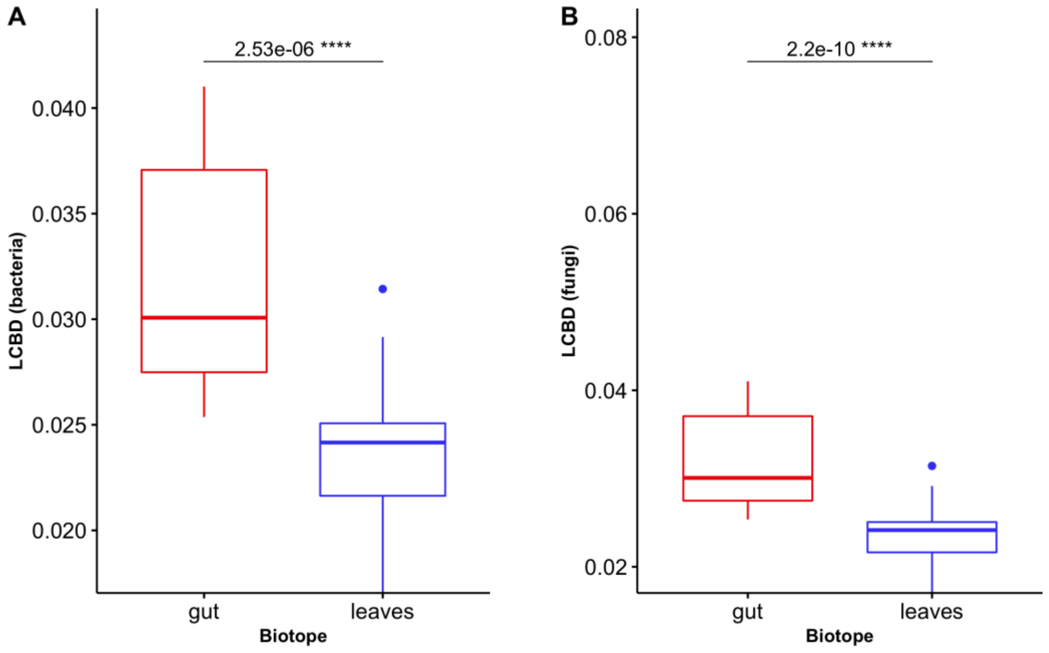

Supp. Fig. S3. Comparison of the LCBD values between the EAB and the leaves microbial communities showing a significant difference between the two communities.

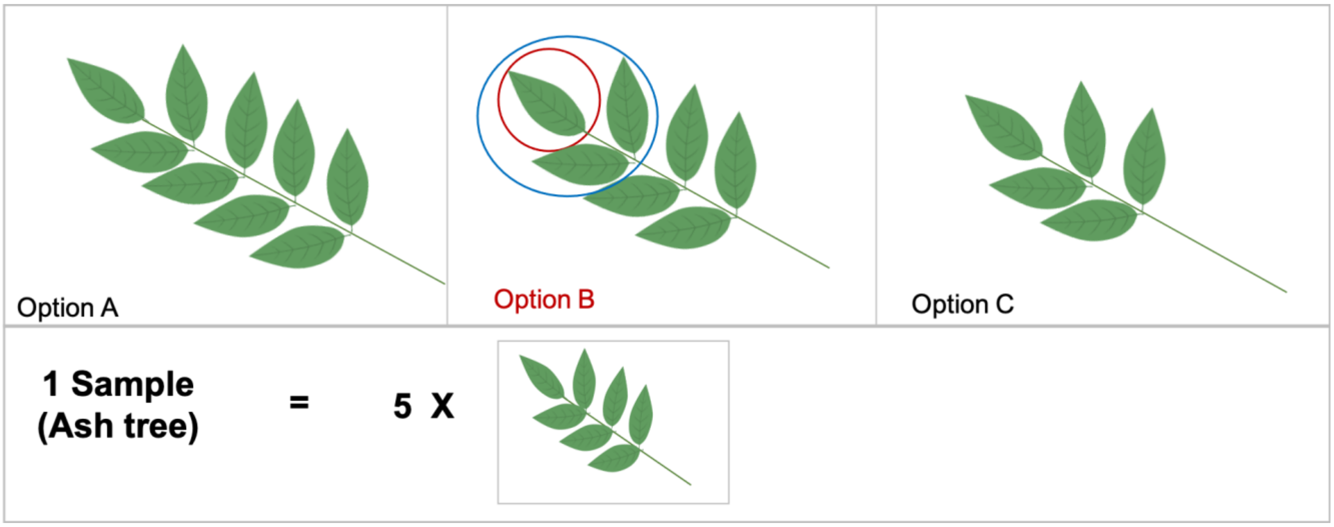

Supp. Fig. S4. For this study the Fraxinus americana was used. Each leaf has five, seven or nine leaflets. For each leaf sample five apical leaflets of five randomly selected leaves were pooled, then used for DNA extraction (in red circle). For each sample the leaflets in the blue circle were used for phytochemicals extraction. During the collection of leaf samples, we obtained three different leaf structures, and we only considered the option B for the sampling.

Table S1. The thirty-three bacterial ASVs assigned at the genus level using Ribosomal Data Base with a confidence level cutoff at 80% identified only in one habitat.

Genus	Phylum	Class	Names	Counts
Only found in the adult EAB gut				
Ewingella	Proteobacteria	Gammaproteobacteria	Asvb49	2462
Cryptococcus	Basidiomycota	Tremellomycetes	Asvf116	1961
Taibaiella	Bacteroidetes	Sphingobacteria	Asvb82	1650
Cordyceps	Ascomycota	Sordariomycetes	Asvf97, Asvf262, Asvf270	1487
Luteibacter	Proteobacteria	Gammaproteobacteria	Asvb63	1434
Coprinellus	Basidiomycota	Agaricomycetes	Asvf158	1036
Pseudochrobactrum	Proteobacteria	Alphaproteobacteria	Asvb129	813
Anoxybacillus	Firmicutes	Bacilli	Asvb233	670
Knoellia	Actinobacteria	Actinobacteria	Asvb283, Asvb494	658
Shewanella	Proteobacteria	Gammaproteobacteria	Asvb224	567
Exiguobacterium	Firmicutes	Bacilli	Asvb407, Asvb561	528
Herbiconiux	Actinobacteria	Actinobacteria	Asvb265	511
Lewia	Ascomycota	Dothideomycetes	Asvf197	499
Gaiella	Actinobacteria	Actinobacteria	Asvb242	498
Rubrobacter	Actinobacteria	Actinobacteria	Asvb324	424
Clostridium_sensu_stricto	Firmicutes	Clostridia	Asvb575, Asvb522	334
Iamia	Actinobacteria	Actinobacteria	Asvb298	332
Pluralibacter	Proteobacteria	Gammaproteobacteria	Asvb209	324
Aquitalea	Proteobacteria	Betaproteobacteria	Asvb347	313
Pelomonas	Proteobacteria	Betaproteobacteria	Asvb270	303
Annulohypoxylon	Ascomycota	Sordariomycetes	Asvf257	298
Methylocaldum	Proteobacteria	Gammaproteobacteria	Asvb460	276
Mesorhizobium	Proteobacteria	Alphaproteobacteria	Asvb498, Asvb674	271
Gemmiger	Firmicutes	Clostridia	Asvb413	225
Globicatella	Firmicutes	Bacilli	Asvb470	215
Morganella	Proteobacteria	Gammaproteobacteria	Asvb430	198
Inocybe	Basidiomycota	Agaricomycetes	Asvf333	184
Leptotrichia	Fusobacteria	Fusobacteria	Asvb532	179
Elizabethkingia	Bacteroidetes	Flavobacteria	Asvb523	173
Arthrobacter	Actinobacteria	Actinobacteria	Asvb459	172
Okibacterium	Actinobacteria	Actinobacteria	Asvb604	172
Actinomyces	Actinobacteria	Actinobacteria	Asvb569	166
Prevotella	Bacteroidetes	Bacteroidia	Asvb529	162
Phanerochaete	Basidiomycota	Agaricomycetes	Asvf407	136
Psathyrella	Basidiomycota	Agaricomycetes	Asvf420	124
Larkinella	Bacteroidetes	Cytophagia	Asvb611	115
Pseudoxanthomonas	Proteobacteria	Gammaproteobacteria	Asvb679	101
Only found in the leaves				
Diaporthe	Ascomycota	Sordariomycetes	Asvf90, Asvf326	1530
Sclerostagonospora	Ascomycota	Dothideomycetes	Asvf168	452
Coniothyrium	Ascomycota	Dothideomycetes	Asvf209	421
Diatrypella	Ascomycota	Sordariomycetes	Asvf352	230
Preussia	Ascomycota	Dothideomycetes	Asvf275	199
Neosetophoma	Ascomycota	Dothideomycetes	Asvf316	185
Drechslera	Ascomycota	Dothideomycetes	Asvf322	158
Ochrocladosporium	Ascomycota	Dothideomycetes	Asvf394	128
Lophodermium	Ascomycota	Leotiomycetes	Asvf410	104

[1] Asvb, ASV assigned to bacteria; Asvf, ASV related to fungi.

Table S2. Summary extracted from the three partial RDA results.

Partial RDA on bacterial community associated with adult EAB guts				
	Selected ASVs	Adj. R^2	F	*p*-value
Bacteria (leaves)	Asvb105<br>Asvb869<br>Asvb78<br>Asvb299<br>Asvb205<br>Asvb5	0.3336	2.335	0.001
Fungi (leaves)	Asvf26<br>Asvf145<br>Asvf235<br>Asvf90<br>Asvf263<br>Asvf173	0.3903	2.707	0.001
Phytochemicals	--	--	--	--
Geographical position	MEM6<br>MEM7	0.1135	2.024	0.002

Partial RDA on fungal community associated with EAB gut				
	Selected ASVs	Adj. R^2	F	*p*-value
Bacteria (leaves)	Asvb52<br>Asvb633<br>Asvb10<br>Asvb740<br>Asvb896<br>Asvb114<br>Asvb503	0.501	3.289	0.001
Fungi (leaves)	Asvf486<br>Asvf292<br>Asvf8<br>Asvf123	0.3252	2.928	0.001
Phytochemicals	--	--	--	--
Geographical position	MEM13<br>MEM14	0.182	2.778	0.002
